# Personal Stories of Young Women in Residential Care: Health-Promoting Strategies and Wellbeing

**DOI:** 10.3390/ijerph192416386

**Published:** 2022-12-07

**Authors:** Mira Aurora Marlow, Rita Sørly, Heli Kyllikki Kaatrakoski

**Affiliations:** 1 Department of Social Studies, Faculty of Social Sciences, University of Stavanger, 4036 Stavanger, Norway; 2 Department of Child Welfare and Social Work, Faculty of Humanities, Social Sciences and Education, UiT The Arctic University of Norway, 9019 Tromsø, Norway

**Keywords:** narratives, residential care, social work, wellbeing, young women

## Abstract

Interdisciplinary social work practice produces and circulates narratives of young women in residential care. The dominant narratives often present negative descriptions of this group, and less attention has been paid to their resistance to these “big stories”. This study’s aim is to illuminate this resistance of young women in residential care and to explore how they narrate their experiences of being children at risk who have become women managing everyday life. This study utilises a narrative approach and includes three selected personal stories: two from the participants and one from the first author’s reflections on resistance. Through contextual analysis at the macro, meso and micro levels, we focus on how personal stories can influence interdisciplinary social work services. We found resistance to dominant narratives on the different levels in the chosen stories. Resistance can create space to reconstruct and renarrate reality together and help understand the meaning and power of storytelling and silence. Participants’ resistance can be a tool to rebalance the power between social work practitioners and service users. Based on this analysis, we suggest that interdisciplinary collaborative social work should emphasise service users’ personal stories to a higher degree and, in this way, increase user participation in residential care.

## 1. Introduction

In 2021, 50,520 children and young people received measures from Child Welfare Services in Norway [1]. Among these, 13,508 were subject to placement measures [1], and 971 were placed in child welfare institutions in 2021 [2]. In 2021, 458 girls and young women were living in child welfare institutions, making them the minority in Norwegian residential care [2]. Several studies have shown that young people in residential care are, in many ways, in marginalised positions in Norwegian society, and this marginalisation starts in the early stages of their lives [3,4,5,6]. Girls and young women in residential care are often diagnosed and treated for depression and anxiety [7], which highlights the necessity of knowing more about their experiences as children at risk prior to and during care and how they manage everyday life as young adults after residential care.

This study’s context involves young women in residential care in Norway. The research initially aimed to shed light on how girls and young women in marginalised positions perceive their pre-residential stories as children at risk and their experiences during and after care. However, the study evolved to include some of the dominant narratives in interdisciplinary social work practice. The dominant stories overlook agency (agency is bound to the resistance of the prevailing norms and values in a given society [8], but it is also something that cannot exist without the opportunity to exercise it [9]) and resistance to challenging experiences and life stories [10]. Resistance here refers to acting against or opposing these dominant narratives [11]. If we reproduce and circulate dominant narratives in social work research and practice, we tend to pay less attention to personal stories. However, what if these stories were told from service users’ perspectives?

This paper explores different context levels in relation to two young women’s personal narratives and the first author’s field notes. We connect the participants’ narratives to the macro, meso and micro levels of context [12]. By using context analysis, we demonstrate that narrating and renarrating are essential parts of empowerment in interdisciplinary social work practice. We explore the stories that our participants reveal about their everyday lives and experiences. Narratives have an impact on both storytellers and listeners, and stories are epistemological tools, as narratives help locate human experiences in time and space [13]. By focusing on narrating as an empowering activity, we hope to inspire social work practitioners to take part in supporting young people to enact agency in health-promoting strategies and wellbeing. We suggest storytelling and story listening as important pillars for interdisciplinary collaborative practice that includes a higher degree of user participation.

The article analyses dominant narratives in interdisciplinary social work and the personal stories expressed (i) by the participating young women, (ii) by the involved researcher and (iii) in the encounters between the participants and the researcher. The narrative approach refers to a unique embodied narrative act of storytelling that is performed (or not) between the interviewer and the participant in social and cultural situations [14,15].

### 1.1. Narrative Approach: Dominant Narratives and Small Stories

We are the stories we tell about ourselves [16]. A complex relationship exists between culture and identity, and identification is an ongoing process that continues throughout life [17]. This study is concerned with narratives that draw from cultural beliefs and practices to further a specific project of self-identifying within available dominant narratives. In line with Fivush [16], we agree that people, through narratives that they take part in or are exposed to, are enrichened through explanatory and evaluative frameworks that weave people, places and events together and create stories that define who we are in time and place and in relation to others. Through multiple acts of storytelling, narratives become accepted or contested as evaluative versions of the past. Stories take on moral perspectives, explaining not only what happened and what it means but also what it should mean [16,18].

Dominant narratives are collective representations of how the world is and should be. They are said to be totalising, hegemonic and controlling stories [19] and represent the majority’s values, frequently silencing and taking for granted the voice of others or of those outside the majority [20]. Dominant narratives create social boundaries between people and help categorise people as “others” or “different” from oneself. One of the dangers of dominant narratives is that they stop us from hearing smaller personal stories that are different but still matter.

Small stories are conversations, diaries, letters, blogs, field notes, autobiographies and everyday small talk. The most common of these in research are parts of interviews or sequences of life stories. Life stories can focus on single episodes in a life or certain aspects of a life story—what Bertaux [21] and Pèrez Prieto [22] defined as “topical life stories”. A discussion between big stories and small stories in narrative research has long continued [17], and researchers have defined big stories as related to interviews and biographies and small stories as based on social interactions in different formal and informal contexts [17]. While research on big stories has focused on individual storytelling as a source of knowledge about the storyteller, research on small stories has focused more on the storytelling act as a situated and context-dependent activity.

Through storytelling, service users and social work practitioners can explore the meanings of the experiences “in certain contexts, in certain times, and with certain others” [23] (p. 209). Service users are invited to tell stories of what has happened to them, and social work practitioners analyse meaning from their narratives together with service users [23]. Social work is, by nature, a narrative practice and profession [23,24].

When selecting the data material for this paper, we included one story from the first author’s field notes, along with the participants’ personal stories. These stories were chosen during the analysis because they highlight resistance to dominant narratives in social work.

### 1.2. A Dominant Story of Young Women in Residential Care

Young people in residential care are marginalised in societies, as they are under the care of the state, not of their parents, and are particularly exposed to poverty, exclusion and discrimination [3]. Young people in the care of the state have often been subjected to emotional and physical deprivation, which can lead to psychological challenges [4,5,6]. Experiences of deprivation can have a longitudinal impact on young people’s lives, which may continue to affect their adult lives [4,25].

Norwegian studies on young people in residential care have indicated that they have poorer quality of life, more mental disorders, worse performance at school, a higher incidence of behavioural difficulties and more frequent substance abuse problems compared to their peers [26,27]. In addition, compared to other young people in Norway, they are often more inactive in education or working life as adults [28]. International studies have shown that residents often have mental illnesses, intellectual or learning difficulties, challenging behaviours [29,30,31], substance misuse and/or self-harming issues [32]. The dominant narratives of young people in residential care are about individuals’ psychological struggles. This can deepen their marginalisation, as the way other people view and address young people has a significant impact on how they see themselves and their feelings of belonging [33]. Gender also has an impact on how the residents are narrated. Jozefiak et al. [34] and Oerbeck et al. [7] stated in their research that girls showed a significantly higher probability of depression and anxiety disorders than boys and that boys had a significantly higher probability of being diagnosed with a severe behavioural disorder. Greger et al. [35] added that girls in residential care reported more maltreatment in their upbringing and that young people in residential care are likelier to have mental disorders than other children in Child Protection Services. A longitudinal study from 1981–1998 included 25 girls and young women (12–16 years old) who had multiple problems, such as being without a permanent place to live and having a history of criminality, drug abuse and significant difficulties adapting to school and home [36]. The dominant stories based on these studies circulate narratives of young people who have several psychological, substance abuse and behavioural problems [30,31,32,33,34,35,36]. Less attention has been paid to young women’s resistance to these “big stories” and how they narrate experiences of being children at risk who have become young adults managing everyday life. This article aims to fill this research gap, as the personal stories told by our participants carry specific knowledge of young women’s health-promoting strategies and wellbeing, expressed as resistance to dominant narratives. Therefore, our research question is how young women with experiences in residential care tell stories of being children at risk who have become young adults managing everyday life.

The structure of the paper is as follows. The introduction includes the narrative approach and a brief overview of a dominant narrative of young women in residential care. In the next section, the materials and methods are presented, followed by the results and discussion. In the end, we offer a conclusion highlighting implications for interdisciplinary social work practice.

## 2. Materials and Methods

### 2.1. Institutional Review Board Statement

The current study was conducted between 2018 and 2019 in Norway and was approved by the Norwegian Centre of Research Data (2018-58745/3/LH) and each research site and municipality. Written consent was collected from each participant, and they were informed of their right to withdraw their consent at any point during the study. Throughout the study, close attention was paid to the ethics of care, as the participants told stories about harmful environments. The participants had the opportunity to take breaks or leave if they wished to do so. After each interview, the participants were asked about their experiences while being interviewed, and they had the opportunity to talk and receive further follow-up if needed.

### 2.2. Recruitment

Recruitment was conducted through private and state-run Norwegian residential care facilities. The study was advertised on social media. The snowball method was used to recruit participants. As young people in residential care are hard to reach, recruitment was facilitated through several gatekeepers [37]. Gaining access to possible participants was challenging, although this is not unusual for studies among hard-to-reach populations [37,38]. This might have affected the potential participants’ willingness to join and their attitudes towards the study. Four participants withdrew from the study before or after the first round of interviews.

### 2.3. Participants

The five participants in this study had diverse ethnic backgrounds and varied reasons why they became residents of residential care in Norway. The young women in this study were between 12 and 16 years old when they were placed in residential care and between 17 and 26 at the time of the interviews. The two interviewees presented in this paper had been involved in psychiatric care wards prior to, during and/or after their stays at the institutions.

### 2.4. The Interviews

The narrative interview method was chosen to elicit free storytelling [39]. The five participants in this research told personal stories of their experiences prior, during and after their placements in residential care. These stories were told in interactions with the first author [40]. The participants first talked uninterrupted while the first author took notes for the second phase, in which questions were asked to deepen the understanding of the participants’ narratives. The transcribed interviews were sent to the participants, and they were provided with the opportunity to make changes. Only three participants read their narratives, and none made any adjustments. All participants were invited to take part in the second round of interviews. For the second interviews, the first author constructed interview questions based on the first narratives to obtain richer data. The interviews were conducted in Norwegian and audio-recorded. Altogether, the study included eight interviews.

### 2.5. Narrative Context Analysis

Narrative performative analysis contains different approaches, with context analysis being one of the more well-known methods in social science research [41,42]. Context enters these stories in complex choreography and moves on several levels. The different contexts exist in the spaces between the narrator and the listener, between the telling and the setting, between the reader and the text and between the history and the culture [41]. Considering interviews allows for emphasising that stories are created in the moment and exist in a vital and vibrant setting [43]. There are no limits to relevant contexts, and researchers must decide what should be excluded or included in the context analysis of stories. By focusing on external contexts, we describe how dominant narratives relate to personal stories. Zilber et al. [44] introduced the following three levels of context: the dominant narratives that underlie and give sense to a particular story (macro level), the collective social field in which one’s life and story evolve (meso level) and the immediate relations in which the narrative is produced (micro level). In the following analysis, we present the stories and then employ these spheres to describe the different approaches to dominant narratives in social work.

## 3. Results: Analysing the Stories

We started the analysis process by reading all the participants’ stories and the first author’s field notes. We sought relevant perspectives connected to health-promoting strategies and wellbeing within each story. We focused on the content of the narratives and found that two of the stories and one field note thematised resistance to dominant narratives in social work. The first story is an excerpt from Ida’s narrative, followed by the first author’s notes. Ida’s narrative is interpreted on a macro level. The second story is an extract from the interview with Mia, which is also followed by the first author’s notes. This narrative is interpreted at the meso level. The last story is the first author’s notes related to an interview that was planned but never took place. The untold story is interpreted on a micro level. To understand resistance as contextual health-promoting strategies and expressions of wellbeing, we need to look at how these young women (i) navigated between the dominant story of being neglected by the parents and showing oneself as a present mother, (ii) resisted a previous dominant story as a young woman with substance abuse and psychological problems in residential care and (iii) balanced between telling one’s story and resisting storytelling.

### 3.1. Ida’s Story: “We Came Home and There Was No Food”

This section involves Ida’s narrative and the first author’s field notes. Ida was 26 years old at the time of the interview. She has three younger siblings, and when they were children, they lived with their drug-addict parents until one day, Ida, at the age of 12, walked to the child protection office and talked about the conditions in which they were living. They were all placed with foster families. At the age of 15, Ida was placed in residential care after several failed foster care placements. Ida described her rejection from her parents as follows in her free narrative that was elicited with an encouragement to tell a story:

*Researcher: I want that you tell me your story. You can start wherever you want and decide what you want to tell and what are the most central events in your life. I will just listen and make some notes for later on. […]*

*Ida: There were a lot of things in my childhood that I didn’t want to experience that were twisted to experience…when we came home and there was no food…we got no food before it was a payday…when it was a payday, we had lots of food for some days, and then, it was like it was all used to pay for drugs, and there was never more again.*

(Ida, 1st interview)

After the interviews, the first author made notes describing her impressions of Ida’s story:

*Ida told her narrative without interruption for over an hour and had her small daughter with her. The description above was repeated during the second interview, which strengthened the feeling that this was an emotional encounter that she remembers well after many years. Ida’s narrative was full of painful memories that painted vivid pictures of child abuse and neglect. I was awed by Ida’s strength and determination, that she had done so well in her life.*

At the macro level, dominant narratives give sense to particular stories. Ida’s narrative and the first author’s field notes are closely related to how a dominant narrative of the emotional and physical deprivation of young women in residential care can develop in interdisciplinary social work practice. It is important to acknowledge that this is a story from a pre-residential time in Ida’s life as a child at risk. This is a narrative from the past and not an ongoing story for Ida anymore. She has moved away from the position of being a child at risk to becoming a young woman taking care of her daughter. By bringing her daughter into the interview situation, Ida shows herself as something different from her parents’ neglect, and she resists the dominant narrative she experienced in her childhood. In Ida’s story, it is as important to listen to what she says as it is to see what she does during the interview. Stories include actions, what we do while we are telling the story. By resisting the dominant narrative of parental neglect, showing herself as a present mother, Ida is expressing health-promoting strategies and wellbeing in the current situation. As social work practitioners, we need to relate and understand the movements away from the dominant story of rejection and neglect towards an empowering, personal story of strength.

### 3.2. Mia’s Story: “I’ve Been Seven Months’ Sober”

The following section presents an excerpt of Mia’s story. She was 17 years old at the time of the first interview and was living by herself at an institution, as she was seen as “psychologically too unstable” to live with other young people or with a foster family. While living at the institution, Mia had developed a substance addiction, and she described how she felt that no one cared about her. About a year later, Mia narrated quite a different personal story:

*Researcher: How did it feel to read your interview text [from the last interview session]?*

*Mia: It was OK to read [my interview], but it was weird to read about my life then, because my life is quite different now.*

*Researcher: Yes? A lot has happened since the interview?*

*Mia: Quite a lot has happened in a year. […] So now, I have moved out of the institution.*

*Researcher: So you live alone now?*

*Mia: No, I live with two friends…It didn’t work that well [at the institution]. I was locked inside, followed [by the staff when outside], and they used a lot of physical coercion. So, I was moved from an institution where [the staff] didn’t do anything to one where they used a lot of coercion.*

*Researcher: Yes, ok. But they just let you to move out?*

*Mia: In the last months, I was placed in the institution based on a voluntary agreement, so I just withdrew my consent [Mia laughs]. […]*

*Researcher: So how it is to live outside the residential care?*

*Mia: It is so much better now. I have been seven months’ sober. Life is good. […]*

*Researcher: What do you wish from the future?*

*Mia: My plan for the future is to study to become a lawyer and work with child law in Child Protection Services or work at an institution.*

*Researcher: Where do you see yourself in 10 years?*

*Mia: In 10 years, I wish to go to university and live closer to my little sister.*

After the interview, the first author wrote field notes about the differences between the first-round and second-round interviews:

*Mia kept returning to the positive changes in her life, which shows that she is ready to move on and leave the past behind. It is remarkable how she has managed to stop using drugs, as she was so deep in addiction the last time. It seems to be that she makes a sharp distinction between the first narrative of herself and the new narration of Mia. The focus during this last interview was on her change from being an injecting drug addict to a thriving young woman who has been sober for seven months, returned to high school and has future plans, hopes and dreams.*

This excerpt from Mia’s story is interpreted at the meso level, where the story relates to Mia’s sociohistorical context. Mia’s story of her empowering process is connected to her resistance to coercive treatment. She also connects her story to having started her education in high school and her dream of becoming a lawyer. Her story shows not only how individual stories are framed and shaped by dominant narratives but also how they relate to their surrounding environment. The struggle for renarrating oneself and demarginalising one’s own story and position shows how Mia has developed health-promoting strategies and expresses wellbeing.

Mia resisted her first narrative about herself as an injecting drug addict and mentally unstable, which resonates with the dominant narratives in research on young people in residential care [28]. Mia’s second-round narrative shows how the narrative self is always in flux and the interviewer can only grasp a glimpse of the multiple selves at the time of the interview. Another important issue is how we, as social work practitioners, interpret the stories told. Do we emphasise the drug addiction and resistance of the acute institution or underline the movement towards Mia’s empowerment process through renarrating her own story? Mia’s renarration of herself as one who has resisted the offered treatment and the institutional care and managed to be sober for seven months took place in the interview setting where the first author had prepared questions based on Mia’s first narrative. The new reality and identity were constructed, and Mia’s previous story and identity were resisted in a space between the narrator and the listener. The first author changed the focus of the interview from Mia’s drug addiction to her agency, and thus resisted the dominant narrative of “damaged” young women in residential care. In this way, space was created for a possible new narration of Mia. This space exists between the history of the dominant narrations of young women in residential care and the culture of storytelling among them: the possibility of renarrating and constructing new identities and realities. This space can be created within the meso level—the sociohistorical context of interdisciplinary social work practice.

### 3.3. First Author’s Story: “Anna No Longer Wants to Participate”

Among the first author’s field notes were observations about an interview that never took place. The first author took notes in the field while waiting for the participants and after the interviews. One of the participants, Anna, withdrew from the study, while the first author was at the residential facilities.

*I had travelled over three hours on buses and ferries and a car. I was exhausted but eager to hear Anna’s story. [On the way to the residential care unit] I was told that Anna’s [participation] could be uncertain. At the house, there were two employees: a man and a woman. Apparently, there is a policy to lock all the doors, as it is an institution, I was told. Even if I wanted to use the toilet, I needed to ask an employee to open it. I wondered what the reason behind this procedure was, as only Anna was living there. The woman employee explained that Anna was going to have an appointment at a psychiatric policlinic, and she is often very exhausted afterwards. Perhaps because of the appointment, Anna was hesitating to participate in the study. I said that I understood that, and if she only wanted to see me or ask some questions without participation, that was fine. […] Anna no longer wanted to participate.*

The micro level reflects the immediate production of a narrative. This excerpt is an inner dialogue from the first author’s field notes. It represents the researcher’s immediate reflections at the micro level of Anna’s untold story. This is interpreted as a resistance of the first author’s presence in Anna’s home, of storytelling and of the research project as a whole. The first author is discussing the act of resistance in a dialogue with herself, and the field notes tell a story of disappointment, exhaustion and rejection. It is not unusual in interdisciplinary social work practice to be rejected by the service user and to feel disappointment. Yet, coping with and tolerating the rejection is necessary. Service users refusing to tell their stories while in social service can be interpreted as a use of power to resist storytelling and a silent resistance to reproducing dominant narratives. A space for the renarration of their own stories needs to be created between the service user and the social work practitioner. What does Anna tell us with her silence, and what does her resistance mean? This yields an understanding of the context of the situation in which the story is constructed. Anna lives in residential care and is described in the first author’s field note as psychologically vulnerable. Anna wanted to participate until the day of the interview but suddenly withdrew from the research. Perhaps she refused to tell the dominant narrative of the “damaged” young women in residential care, and in this way, resisted storytelling. The first author interprets that the distance between Anna and her was growing larger. Similarly, in social work, the respect and recognition of silent resistance, a language of untold stories, is vital. Untold stories also have a function in interdisciplinary social work practice that shows the need to respect and give space to service users and understand what silence means. Silence can also protect service users and be an expression of health-promoting strategies in terms of taking care of themselves.

## 4. Discussion: Personal Stories as Health-Promoting Strategies and Wellbeing

In this paper, we studied how young women with experiences in residential care tell stories about their progression from being children at risk to becoming young adults managing everyday life. Resistance to dominant narratives was interpreted from their personal stories. Our study revealed resistance as a narrative tool to (i) rebalance the power between social work practitioners and service users, (ii) create space to reconstruct and renarrate reality together and (iii) help understand the meaning and power of storytelling and silence.

Some of these findings were identified in the following studies: a study examining the distance in social work created when practitioners silenced the service users’ views and/or degraded their experiences [45], causing everyday resistance to emerge; an examination of how homeless service users talked back or refused to talk when feeling that their views or experiences were not respected [46]; a study of service users who identified as 2SLGBTQ (Two-Spirit, lesbian, gay, bisexual, transgender, queer, or questioning) with experiences of psychosis resisted unjust treatment with “anger/non-compliance/disagreement” (p. 189) and with forms of quiet resistance [47]; and an examination of young women in residential care in India who resisted pathologising the dominant narratives of their lives [48].

However, little research has been conducted on how narrative social work practice can increase user involvement among young women in residential care. Additionally, less attention has been paid to how they narrate experiences about their progression from being children at risk to becoming young women managing everyday life, highlighting the changes in their personal stories. We believe that the personal stories told by our participants carry specific knowledge of young women’s health-promoting strategies and wellbeing, expressed as resistance to dominant narratives. We found resistance to dominant narratives at the different levels in the chosen stories. These were expressed at the following levels:

Macro level—resisting the dominant narrative of parental neglect;

Meso level—resisting the dominant narrative of young women in residential care;

Micro level—resistance as a way of protecting oneself.

These levels are illuminated in the following sections.

### 4.1. Macro Level: Resisting Dominant Narratives

Service users’ resistance to dominant narratives must be acknowledged in social work practice [11,48]. Practitioners need to consider the change within personal stories from being a child at risk to becoming young people managing everyday life [48]. The dilemma here is that if practitioners reproduce the dominant narratives, they can contribute to service users’ marginalisation, but if they are resistant to reproducing the dominant stories, practitioners are in a position to decide which stories are told and which are left aside. If the painful narratives of the participants’ lives are ignored, does this mean that these stories are not as important as the narratives of resistance, agency or empowerment? There are always some stories that are not (re)presented or (re)produced. Who has the right to decide what is important to tell?

### 4.2. Meso Level: Resisting Previous Narratives

The meso-level personal stories in this study revealed the importance of giving service users space to move from the previous negative life narration towards the positive. By emphasising the positive changes in their lives and personal stories, they help provide opportunities for service users to adjust the course of their lives and reconstruct their identities. In interdisciplinary social work practice, it is necessary to create space for positive development and have healthy expectations in abnormal conditions [49]. As practitioners, we need to resist reproducing dominant narratives and predefinitions of young women in residential care [48] and instead take part in an ongoing dialogue of narration and renarration. Thus, we ask who has the right to label service users who are already in marginalised positions in society? Should the focus instead be on service users’ participation and involvement in their own care and individualised treatment?

### 4.3. Micro Level: Resisting the Research and Silence

In line with Godsil and Goodale [50], we agree that the stories that we tell ourselves can strongly shape our behaviour. The hopeful possibility is that by reshaping our narratives, we can change our behaviour and ultimately our outcomes [50]. The untold story shows how important it is to respect and give space to service users and to understand the power of silence [51], thus creating room for possible future storytelling practice in interdisciplinary social work practice. Silence can indicate a lack of mutual language and trust between service users and practitioners [47], which limits the possibilities of creating new narratives and a new reality together in interdisciplinary social work practice. Silence and refusal to take part in storytelling are also context-bounded and related to not reproducing the dominant narratives of service users [52]. This can challenge the power relations between service users and practitioners [52,53]. Narrative practice can help find a mutual language and build trust between practitioners and service users and can be a starting point for creating a new reality together. The untold story reflects a well-known dilemma in interdisciplinary social work practice [52], but perhaps the silence itself is not the problem, but rather how silence and resistance are treated and understood in social work.

### 4.4. Understanding Resistance

In the first author’s field notes, she sought to understand why and how she was rejected. Perhaps rejection was the participant’s way of using silence as power. Any strategies of secrecy and nonconscious silences are shaped by the individual’s biography, social relations, hopes, anxieties and aspirations [54]. From the point of view of interdisciplinary social work practice, we interpret resistance here as a way to equalise the power relations between service users and practitioners. To tell a personal story, service users need to have respect from and a mutual language with the practitioners. There also needs to be a safe space to create a new reality and to coconstruct new stories.

### 4.5. Limitations of the Study

The present study has several limitations. First, as in all qualitative studies, the results cannot be generalised due to the small number of participants in the research. Instead, this study provides insight into personal experiences from these young women’s points of view in Norwegian residential care. Second, the study focused only on young women’s experiences and did not present young men’s or social workers’ perspectives on Norwegian residential care.

## 5. Conclusions

The findings presented in this article have the following practical implications to social work practice. Practitioners can benefit from focusing on how young women with history in residential care tell, or refuse to tell, stories about being children at risk who have become young adults managing everyday life, as this will help social work practitioners better understand how service users resist negative dominant stories of them. Furthermore, narrative social work practice acknowledges that while service users tell or not tell stories, they also act to promote strategies of wellbeing. Wellbeing is closely connected to social justice and human rights. In line with Baldwin [54], we accept narratives as parts of social work practice, human rights and social justice. It is not enough to work with narratives of service users for social change, but as practitioners, we need to become part of these stories on equal terms. Social work practitioners must take service users’ participatory and human rights and their best interests seriously. This is especially necessary in the residential care of children and young people, as participation in their own care is highlighted in the new Norwegian Child Welfare Act [55]. Future research must investigate the possibilities of narrative social work practice and how it can increase user participation in the residential care of young people.

## Data Availability

The data are not publicly available due the ethical considerations; to secure the privacy of the participants; to protect their anonymity; and due the sensitive information.

## References

[1] Statistics Norway (2022). Child Welfare. https://www.ssb.no/en/sosiale-forhold-og-kriminalitet/barne-og-familievern/statistikk/barnevern.

[2] Bufdir Children and Young People in Institutions. https://bufdir.no/Statistikk_og_analyse/Barnevern/Barn_og_unge_med_tiltak_fra_barnevernet/barn_i_institusjon/.

[3] Council of Europe Strategy for the Rights of the Child (2022–2027). https://rm.coe.int/council-of-europe-strategy-for-the-rights-of-the-child-2022-2027-child/1680a5ef27.

[4] Eltink E.M.A., ten Hoeve J., de Jongh T., van der Helm G.H.P., Wissink I.B., Stams G. (2018). Stability and change of adolescents’ aggressive behavior in residential youth care. Child Youth Care Forum.

[5] Jozefiak T., Kayed N.S., Ranøyen I., Greger H.K., Wallander J.L., Wichstrøm L. (2017). Quality of life among adolescents living in residential youth care: Do domain-specific self-esteem and psychopathology contribute?. Qual. Life Res..

[6] Magruder K.M., McLaughlin K.A., Elmore Borbon D.L. (2017). Trauma is a public health issue. Eur. J. Psychotraumatol..

[7] Oerbeck B., Overgaard K.R., Hjellvik V., Lien L., Bramness J.G. (2021). The use of antidepressants, antipsychotics, and stimulants in youth residential care. J. Child Adolesc. Psychopharmacol..

[8] Basnyat I., Dutta M.J., Zapata D.B. (2019). Self-reflexivity for social change: The researcher, I, and the researched, female street-based commercial sex workers, gendered contexts. Communicating for Social Change: Meaning, Power, and Resistance.

[9] Abebe T. (2019). Reconceptualising children’s agency as continuum and interdependence. Soc. Sci..

[10] Marlow M.A., Gunnarsdottir H.M., Studsrød I. (2022). “No one saw us, and no one did anything”: Marginalised young women narrating management of (in)visibility and intersecting identities.

[11] Weinberg M., Banks S. (2019). Practising ethically in unethical times: Everyday resistance in social work. Ethics Soc. Welf..

[12] Serpa S., Ferreira C.M. (2019). Micro, meso and macro levels of social analysis. Int. J. Soc. Sci..

[13] Brown P. (2017). Narrative: An ontology, epistemology and methodology for pro-environmental psychology research. Energy Res. Soc. Sci..

[14] Ellingson L.L. (2017). Embodiment in Qualitative Research.

[15] Svahn J., Sørly R., Blix B.H. (2017). “Jag har bara ADHD å så”: Identitetskonstruktion och normalisering i resursskoleelevers berättelser. Fortelling og forskning: Narrativ Teori og Metode i Tverrfaglig Perspektiv.

[16] Fivush R. (2010). Speaking silence: The social construction of silence in autobiographical and cultural narratives. Memory.

[17] Blix B.H., Hamran T., Normann H.K. (2013). Struggles of being and becoming: A dialogical narrative analysis of the life stories of Sami elderly. J. Aging Stud..

[18] Sørly R., Mathisen V., Kvernmo S. (2021). “We belong to nature”: Communicating mental health in an indigenous context. Qual. Soc. Work.

[19] Linde C. (2009). Working the Past: Narrative and Institutional Memory.

[20] Mishler E.G. (1995). Models of narrative analysis: A typology. J. Narrat. Life Hist..

[21] Bertaux D., Bertaux D. (1981). Introduction, biography and society: The life history approach in the social sciences. Biography and Society: The Life History Approach in the Social Sciences.

[22] Pérez Prieto H. (2006). Historien om Räven och Andra Berättelser: Om Klasskamrater och Skolan på en Liten ort—Ur ett skol-och Livsberättelse Perspektiv. Karlstad Univ. Stud..

[23] Shaw J. (2017). Thinking with stories: A renewed call for narrative inquiry as a social work epistemology and methodology. Can. Soc. Work Rev..

[24] Baldwin C. (2013). Narrative Social Work: Theory and Application.

[25] Greger H.K., Myhre A.K., Klöckner C.A., Jozefiak T. (2017). Childhood maltreatment, psychopathology and well-being: The mediator role of global self-esteem, attachment difficulties and substance use. Child Abus. Negl..

[26] Singstad M.T., Wallander J.L., Greger H.K., Lydersen S., Kayed N.S. (2021). Perceived social support and quality of life among adolescents in residential youth care: A cross-sectional study. Health Qual. Life Outcomes.

[27] Backe-Hansen E., Løvgren M., Neumann C.E.B., Storø J. (2017). God Omsorg i Barnevernsinstitusjoner.

[28] Nordahl K.B., Sørlie M.A. (2021). Ny kunnskap om barn i kontakt med barnevernet og psykisk helsevern for barn og unge. Tidsskr. Nor. Barnevern.

[29] Bath H. (2008). Residential care in Australia, Part I: Service trends, the young people in care, and needs-based responses. Child Aust..

[30] Swerts C., De Maeyer J., Lombardi M., Waterschoot I., Vanderplasschen W., Claes C. (2019). “You shouldn’t look at us strangely”: An exploratory study on personal perspectives on quality of life of adolescents with emotional and behavioral disorders in residential youth care. Appl. Res. Qual. Life.

[31] Clemens V., Bürgin D., Eckert A., Kind N., Dölitzsch C., Fegert J.M., Schmid M. (2020). Hypothalamic-pituitary-adrenal axis activation in a high-risk sample of children, adolescents and young adults in residential youth care—Associations with adverse childhood experiences and mental health problems. Psychiatry Res..

[32] McPherson L., Gatwiri K., Tucci J., Mitchell J., Macnamara N. (2018). A paradigm shift in responding to children who have experienced trauma: The Australian treatment and care for kids program. Child. Youth Serv. Rev..

[33] Heng L., White J., Guerin A. (2018). Employing intersectionality and the concept of difference to investigate belonging and inclusion. Belonging: Rethinking Inclusive Practices to Support Well-Being and Identity.

[34] Jozefiak T., Kayed N.S., Rimehaug T., Wormdal A.K., Brubakk A.M., Wichstrøm L. (2016). Prevalence and comorbidity of mental disorders among adolescents living in residential youth care. Eur. Child Adolesc. Psychiatry.

[35] Greger H.K., Myhre A.K., Lydersen S., Jozefiak T. (2015). Previous maltreatment and present mental health in a high-risk adolescent population. Child Abus. Negl..

[36] Helgeland I.M. (2007). Unge Med Atferdsvansker Blir Voksne: Hvordan Kommer de inn i et Positivt Spor?.

[37] Abrams L.S. (2010). Sampling “hard to reach” populations in qualitative research: The case of incarcerated youth. Qual. Soc. Work.

[38] Ellard-Gray A., Jeffrey N.K., Choubak M., Crann S.E. (2015). Finding the hidden participant: Solutions for recruiting hidden, hard-to-reach, and vulnerable populations. Int. J. Qual. Methods.

[39] Thornhill H., Clare L., May R. (2004). Escape, enlightenment and endurance: Narratives of recovery from psychosis. Anthr. Med..

[40] Røkkum N.H.A., Kojan B.H. (2020). Exploring normative complexity: (How) does the life history interview work in child welfare research?. Qual. Soc. Work.

[41] Riessman C.K. (2008). Narrative Methods for the Human Sciences.

[42] Bengtsson T.T., Andersen D., Järvinen M., Mik-Meyer N. (2020). Narrative analysis: Thematic, structural and performative. Qualitative Analysis: Eight Approaches for the Social Sciences.

[43] Klausen R.K., Haugsgjerd S., Lorem G.F. (2013). “The lady in the coffin”—Delusions and hearing voices: A narrative performance of insight. Qual. Inq..

[44] Zilber T.B., Tuval-Mashiach R., Lieblich A. (2008). The embedded narrative: Navigating through multiple contexts. Qual. Inq..

[45] Lee E. (2022). Fact construction and categorization in assessment: Cultivating epistemic justice and resistance in social work assessment. Soc. Serv. Rev..

[46] Mik-Meyer N., Haugaard M. (2021). Performance of agency in real-life encounters: Turning unequal power and structural constraint into collaboration. Symb. Interact..

[47] de Bie A., Daley A., Ross L.E., Kidd S.A. (2021). Resisting unmet expectations as service user ethics: Implications for social work. J. Prog. Hum. Serv..

[48] Sen M. (2019). Working with young people in residential care in India: Uncovering stories of resistance. Int. J. Narrat. Community Work.

[49] Cameron-Mathiassen J., Leiper J., Simpson J., McDermott E. (2022). What was care like for me? A systematic review of the experiences of young people living in residential care. Child. Youth Serv. Rev..

[50] Godsil R.D., Goodale B. (2013). Telling our own story: The role of narrative in racial healing. Am. Values Inst..

[51] Khalid A. (2022). The negotiations of Pakistani mothers’ agency with structure: Towards a research practice of hearing “silences” as a strategy. Gend. Educ..

[52] Brown B.S. (2020). Stories from the Inside: An Exploration into Prisoner Identity, Narrative, and the Violence of Silence. Master’s Thesis.

[53] Revell L. (2022). Narratives of neglect in social work with children and families: The relationship between voice and narrative. Child Abus. Rev..

[54] Samuels A. (2021). Strategies of silence in an age of transparency: Navigating HIV and visibility in Aceh, Indonesia. Hist. Anthropol..

[55] NRDCF (2021). Prop. 133 L (2020–2021). Regejeringen. https://www.regjeringen.no/no/dokumenter/prop.-133-l-20202021/id2842271/.

